# Maltine with Creosote in Infectious Diseases

**Published:** 1899-08

**Authors:** 


### Selections and Abstracts.

### MALTINE WITH CREOSOTE IN INFECTIOUS DISEASES.

THE study of infectious disease received a new impetus and was placed upon a new basis when the agency of bacteria in its production was discovered. The efforts of clinicians were then directed to the influence of remedies upon the parasitic and living causes of disease. The great desideratum was to find substances having the power either to destroy microbes or to neutralise the noxious products which they elaborate. In the course of such experimental studies, however, we were led to realise more forcibly than ever before the resistant powers of the human organism. It was learned that it is not the mere presence of bacteria within the body that is the most significant fact, but their germination, reproduction and cultivation, and, above all, the poisonous products by which the infection of blood and tissues is accomplished. Thereafter the fortification of the organism acquired fresh importance. The attention of physicians was directed not only to the destruction of microörganisms and the neutralisation of their poisons, or toxins but also to the assistance of the tissues in their struggle against the invaders.

As long as, by any and every means, general nutrition can be maintained at the normal standard there is little to be feared from the presence of pathogenic bacteria. If, however, the general vitality be reduced by any cause, our diminutive foes can then not only enter, but can contaminate the system.

These discoveries have thrown new light upon the operation of many medicinal substances, and have served to direct our energies to the support of the threatened organs and tissues. A nutritious principle which is so influential in promoting the digestion of one of the great food-groups—viz.: the carbohydrates—has a wide range of applicability. It adds to the nourishment of the feeble. It restores digestive power and physical energy to those who have been notably reduced by a lingering illness. It promotes the healthy growth of muscular structures and strengthens the functions of secreting glands.

Accordingly skilfully prepared and reliable preparations have long been favorably known to and beneficially employed by physicians in the large class of morbid conditions in which they are indicated. Several active remedies or combination of remedies have from time to time been added to the plain maltine in order to adapt it to a wider field of usefulness. The latest of these excellent additions to a worthy line of products is maltine with creosote.

In the purely medicinal, as distinguished from the climatic treatment of tuberculosis, creosote has approved itself as a remedy of the first rank. It undoubtedly possesses considerable inhibitory influence over the development of the bacillus tuberculosis. It relieves the prominent symptoms of phthisis more effectually than any other remedy. Creosote is often able to hold this destructive malady in abeyance for an indefinite period or practically cure the disease. Therefore a combination of maltine with creosote appeals most powerfully to the medical profession. So much of the physician's work has to do with tuberculosis in its varied manifestations and localisations that a warm welcome will doubtless be extended to this new preparation. Its nutrient and antiseptic properties render it admirably adapted to fulfill many important indications. We are assured that the creosote contained in this preparation is perfectly pure, and we have learned by painful experience that there is a vast difference between pure and impure creosote. Each fluid ounce of maltine with creosote contains 4 minims of pure creosote. Creosote is an efficient remedy in many morbid conditions of the intestinal tract, and this new combination will, consequently, be found of service in many cases of chronic indigestion.—*Monthly Cyclopedia of Practical Medicine*, June, 1899.

